# Dose-Dependent Transient Decrease of Impedances by Deep Intracochlear Injection of Triamcinolone With a Cochlear Catheter Prior to Cochlear Implantation–1 Year Data

**DOI:** 10.3389/fneur.2020.00258

**Published:** 2020-04-15

**Authors:** Nils K. Prenzler, Rolf Salcher, Thomas Lenarz, Lutz Gaertner, Athanasia Warnecke

**Affiliations:** ^1^Department of Otorhinolaryngology, Head and Neck Surgery, Hanover Medical School, Hanover, Germany; ^2^Cluster of Excellence “Hearing4all2.0” of the German Research Foundation, Hanover, Germany

**Keywords:** cochlear implant, impedances, steroids, catheter, drug delivery, inner ear

## Abstract

Administration of low-dose steroids via a catheter inserted into the cochlea to apply pharmaceuticals to more apical regions was previously shown not to be sufficient for long-term reduction of electrode impedances. The aim of the present study was to investigate the effect of intra-cochlear high-dose triamcinolone application on impedances in cochlear implant recipients. Patients received low-dose (4 mg/ml; *n* = 5) or high-dose (20 mg/ml; *n* = 5) triamcinolone via a cochlear catheter just prior to the insertion of a Med-El Flex28 electrode. Impedances were measured at defined time points from intra-operatively up to 12 months after first fitting and retrospectively compared with a control group (no steroid application). Patients who received a high-dose application of crystalloid triamcinolone showed significantly reduced impedances in the first fitting measurements compared to the control group. This effect was no longer detectable in patients of the low-dose group at that time. Looking at the different regions of the electrode, the impedance values were lowered significantly only at the basal and medial contacts. At later time points, there were no significant differences between any of the groups. This is the first study to demonstrate a dose-dependent reduction of impedances by deep intra-cochlear injection of triamcinolone in cochlear implant patients. With a high-dose, single application of triamcinolone using a cochlear catheter prior to insertion of a Flex28 electrode, the impedances can be significantly reduced up to and including the first fitting. Although the effect was longer lasting than when compared to low-dose triamcinolone, it was also not permanent.

## Introduction

The protection of the fine intra-cochlear structures including their neuronal connections for sustained preservation of residual hearing is one major goal in the field of cochlear implantation. Residual hearing is at risk not only during the actual insertion of the CI electrode array, but also in the postoperative course and thereafter (1, 2). There are several approaches to reduce insertion trauma: in addition to less traumatic electrodes and a slow, gentle insertion technique, pharmacological approaches are gaining more and more attention (3, 4). A review article written by Nguyen and colleagues emphasize the use of anti-inflammatory drugs to minimize trauma and inflammation, thereby preserving residual hearing (4). They however conclude that neither dosage nor timing of the application is standardized and still discussed controversially. Short-term efficacy of single-dose transtympanic steroid application prior to cochlear implantation has been proven successfully in a randomized controlled trial involving 30 cochlear implant patients (5). In the longer term, however, only minimal efficacy was shown (5). Other studies have therefore concentrated on the use of prolonged steroid therapy using combined oral and intravenous steroids to stabilize and preserved hearing in cochlear implantation (6). Another approach proven more effective than single-dose steroids was the use of a perioperative oral steroid taper on low frequency hearing preservation (7). Hence, it has already been shown clinically that a perioperative steroid application has, to some extend, a positive effect on residual, low frequency hearing (5–8).

In addition to immediate mechanical cochlear damage during insertion, inflammatory processes and growth of connective tissue intra-cochlearly are the main hallmarks that are thought to lead to loss of residual hearing (9, 10). Animal studies have shown that locally administered steroids reduce the connective tissue growth around the electrode array and that this reduction is reflected by reduced impedances measured over the electrode contacts (11, 12). It is also assumed that acute intra-cochlear inflammation, causing for example dizziness, hearing loss or tinnitus, can result in transient or permanent elevation of impedance levels after cochlear implantation (2, 13, 14).

Systemic application of steroids inherits the disadvantage of unwanted adverse effects and ineffective accumulation of therapeutic drug concentrations inside the cochlea (15). Furthermore, there is no evidence that systemic steroids are effective for the reduction of impedances in human cochlear implant recipients (16). Alternative approaches include local transtympanic administration, allowing the drug to diffuse through the round window into the cochlea. Anatomical variations, varying permeability of the round window membrane and poor diffusion patterns within the cochlea might result in an insufficient dose accumulation of the steroids with lack of effect (14). Attempts to enhance absorption and distribution of dexamethasone to the inner ear after transtympanic application at the round window levels have been successfully demonstrated in an animal model by the use of hyaluronic acid and histamine (17). Another approach to increase steroid concentrations in the inner ear is the direct intracochlear application. After intra-cochlear administration of triamcinolone into the basal turn of the cochlea (manual injection at the site of the cochleostomy) prior to the insertion of a Nucleus Contour Advanced® electrode, the long lasting decrease of impedance values could only be detected in the basal region of the cochlea (18). To obtain any anti-fibrotic effects of the steroid also in medial and apical regions, a cochlear catheter was developed that allows a deeper intra-cochlear application of fluids prior to electrode insertion (17, 18). In a safety and feasibility study from our group, it was possible to achieve a transient decrease of the impedances across all regions of the cochlea (basal, medial, and apical) with an intra-cochlear administration of triamcinolone with a cochlear catheter deeply inserted into the cochlea, whereby no adverse effects were observed in any of the patients (19). Despite initial reduction of impedances for up to 17 days, there was no significant difference of the impedance values between the treatment and the control group thereafter (19). A possible reason could be the lower concentration of steroids that was applied: 4 mg/ml compared to 20 mg/ml in Paasche et al., where a long-lasting reduction of impedances was achieved in the basal regions of the cochlea (18). Based on these observations, we changed our treatment regimen by applying a higher dose of triamcinolon (20 mg/ml) via the cochlear catheter to patients during cochlear implantation. The present retrospective analysis of these cases aims to clarify whether a more pronounced and/or longer lasting decrease of impedance values can be achieved by increasing the dosage of triamcinolon.

## Materials and Methods

### Cochlear Catheter

For a detailed description of the catheter and the intraoperative handling, we refer to our previous publication (19). Briefly, the silicone catheter is a custom-made device from Med-El (Innsbruck, Austria) manufactured from the same silicone as the cochlear implant electrode arrays. It consists of a reservoir at the back end, a tube and a 20 mm long tip with a fluid outlet for the drug to be applied. The inserted part corresponds in its diameter to the proximal 20 mm of the Med-El standard electrode (i.e., starting with 1.3 mm at the base, narrowing to 0.8 mm within the next 6 mm and keeping this diameter thereafter). The tip outlet has a diameter of 0.3 mm. Directly before insertion, the round window membrane was incised crosswise. Then, the catheter was fully inserted (20 mm into the cochlea). Very slowly and via a 1 ml syringe connected to the catheter, the steroid suspension was injected into the cochlea until a return of the milky substance could be observed at the round window. The speed of injection cannot be objectified and was subject to the surgeon's experience. The catheter was then retracted very slowly with further injection, discarded, and then the Flex28 electrode was inserted using our standard technique.

### Study Design and Patients

This study presents the retrospective analysis of 5 patients receiving high-dose steroids via an intracochlear catheter. The results were compared with the study and the control group of a previous clinical investigation. All patients included in the present retrospective analysis had received intra-cochlear triamcinolone injections via the cochlear catheter after fulfilling the following preoperatively evaluated criteria: age at least 18 years, hearing loss >60 dB at 500 Hz, regular anatomical of the temporal bones as evaluated by CT and MRI scans, as well as the choice to receive the Med-El implant with a Flex28 electrode. Patients treated with the catheter have been extensively informed about the opportunities and risks and have deliberately opted for this procedure. Written informed consent was obtained from all participants. The use of this custom-made device as well as the performance of electrophysiological measurements was reported to and approved by the local ethics committee (Hannover Medical School, reference numbers 2740-2015 and 3279-2016). The data collected were formally anonymized and evaluated retrospectively.

Five patients (28–83 years old, 1 female, 4 male) received a deep intra-cochlear administration of triamcinolone at a concentration of 20 mg/ml (“high-dose”) prior to insertion of the Flex28 electrode array. These patients were compared to the patients from our first study (37–75 years old, 4 female, 1 male), who received a deep intra-cochlear administration of triamcinolone at a concentration of 4 mg/ml (“low-dose”) prior to insertion of the Flex28 electrode (19). Both groups were also compared to a control group (40–81 years old, 1 female, 4 male) (19). The control group did not receive any steroids. placebo injections or a catheter insertion.

### Impedance Measurements

Electrode impedances were obtained using the standard Med-El telemetry system (MAX interface box connected to a personal computer, clinical software Maestro 6 and Maestro 7) to perform impedance field telemetry (IFT) measurements on all 12 electrode contacts.

The impedance measurements were performed at standard time points: intra-operatively, test tone (1–4 days postoperatively), first fitting before (FF) and after (FF-el) chronic electrical stimulation, as well as at 3, 6, and 12 months thereafter. The low dose and the control group from a previous study were additionally measured on days 10, 17, and 24. The first fitting of the device was initiated around 5 weeks after surgery and was completed within 1 week in our clinic. Until the first fitting, there was no electrical stimulation of the cochlea (i.e., no audio processor was worn).

To perform the IFT under comparable conditions, the measurement protocol included a short time of electrical stimulation prior to impedance measurement. The IFT were performed after measurements of the electrically evoked compound action potential (ECAP) and stapedius reflex measurements intraoperatively, as well as after ECAP measurements at the test tone. During the first fitting week, the IFT values were measured before chronic electrical stimulation (FF) as well as on the third day of the fitting week (FF-el).

### Statistical Analysis

Impedance measurements of all 12 electrode contacts were averaged per patient for every time point for each impedance value. To assess different regions of the cochlea, the electrode contacts were furthermore clustered and averaged as follows: apical (C1–C5), medial (C6–C8), and basal (C9–C12). The clustering of the electrode contacts was accounting not only due to the different regions that are covered intra-cochlearly but also the fact that C1–C5 in the Flex28 electrode array are single (on one side) and C6–C12 are double electrode contacts (on both sides). Thus, contact C5 was added to the apical contacts, since the single sided contacts might *per se* exhibit different impedance values.

The three groups (low dose, high dose, controls) were treated as independent samples. To test for equality of variances, the Levene's test was used. One-way analysis of variance (ANOVA) was used to test for significant differences among the groups. Since Levene's test for equality of variances was significant (except for overall, apical and medial contacts at the time point 3 days after surgery), the Tukey *post-hoc* test was applied. *P* < 0.05 were considered significant. All data were analyzed statistically using IBM SPSS Statistics 22.

## Results

None of the patients enrolled in this analysis experienced any perioperative complications. The correct placement of the electrode array inside the cochlea/scala tympani was verified by performing cone beam CT scans after implantation. Since the groups were small and heterogenic regarding age and duration of deafness, no statistical comparison of the functional results was performed.

### Impedance Measurements

**Figure 1:**
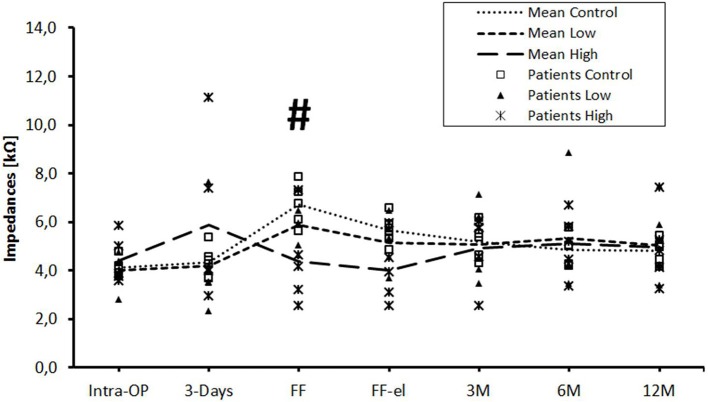
Change in mean impedance values over time for all electrode contacts C1–C12. Significant differences between high-dose and control groups are marked by a hashtag (^#^*p* < 0.05). There were no significant differences between low-dose and control groups or between low-dose and high-dose groups.

In the control group, impedance values for contacts 1–12 increased between day 3 after surgery until first fitting (Figure 1). After initial activation, impedance values decreased immediately (FF-el). At the 3 months appointment, impedances had further decreased slightly. Afterwards, up to the 12 months re-fitting, impedances stayed relatively stable in the control group.

Patients of the low-dose triamcinolone group showed slightly lower impedances at initial activation before and after electric stimulation compared to controls. Differences were not statistically significant. This effect was completely missing at the 3 months appointment. From there on, the course of mean impedance values stayed very similar to the control group.

Mean impedance values of the high-dose group were higher 3 days after implantation compared to controls (*p* = *ns*). Over the course of time, values dropped until first fitting and impedances were significantly lower compared to controls [before (*p* = 0.047) stimulation]. The significant difference between the high-dose and the control group was still present but not significant after stimulation (*p* = 0.072). Using the Tukey *post-hoc* test, there was a significant different between the high-dose and the control group at first fitting before electrical stimulation (*p* = 0.041), but not after stimulation (*p* = 0.072). From the 3 months appointment on, no differences were seen between the groups. There were no significant differences between the low- and the high-dose groups at any time point.

**Figure 2:**
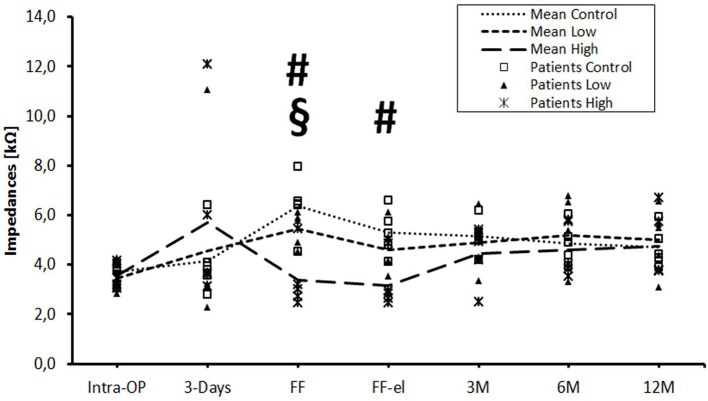
Change in mean impedance values over time for basal electrode contacts C9–C12. Significant differences between high-dose and control groups are marked by a hashtag (^#^*p* < 0.05). Significant differences between high-dose and low-dose groups are marked by a paragraph sign (^§^*p* < 0.05). There were no significant differences between low-dose and control groups.

**Figure 3:**
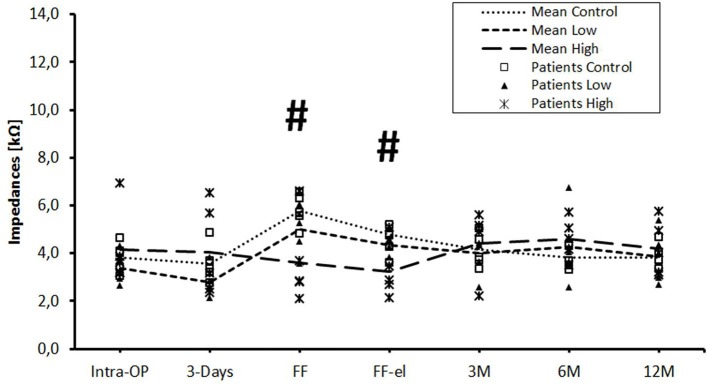
Change in mean impedance values over time for medial electrode contacts C6–C8. Significant differences between high-dose and control groups are marked by a hashtag (^#^*p* < 0.05). There were no significant differences between low-dose and control groups or between low-dose and high-dose groups.

**Figure 4:**
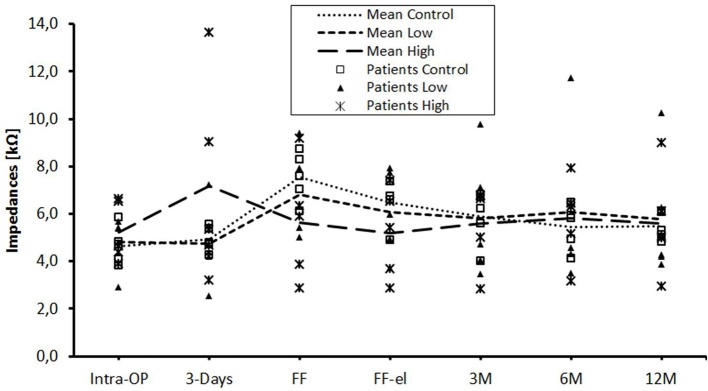
Change in mean impedance values over time for apical electrode contacts C1–C5. There were no significant differences between any of the groups.

The data were also analyzed individually for the different regions of the cochlea. When considering the basal (Figure 2), medial (Figure 3), and apical (Figure 4) contact groups, the course of mean impedance values resembles the course of the entire electrode (Figure 1). Significant differences between controls and the high-dose group were only found at first fitting before and after stimulation at basal (FF day 1: *p* = 0.002; FF day 3: *p* = 0.012) and medial (FF day 1: p: 0.042; FF day 3: *p* = 0.013) contact groups (Tukey *post-hoc* test).

The only statistically significant difference between the low- and the high-dose group was found at day 1 of the first fitting on basal contacts (*p* = 0.024).

## Discussion

In the present study, a dose-dependent reduction of impedances due to an intra-cochlear triamcinolone application via a cochlear catheter has been demonstrated. The time course of impedance reduction varied depending on the applied concentration. The maximum reduction that was achieved by high-dose triamcinolone was observed at the first fitting and this time point equals ~4–6 weeks after steroid application. In a previous study however, the maximum of impedance reduction was observed at approximately 10 days after steroid application when low-dose triamcinolone was administered. Paasche et al. applied a single intracochlear injection of triamcinolone acetonide at a higher concentration of 20 mg/ml using a cannula at the basal turn of the cochlear leading to a pronounced and sustained decrease of impedances over months mainly at the basal electrode contacts (18, 20). To achieve this effect more apically, the catheter described above was developed. The Flex28 electrodes of Med-El are well-suited for such investigations because they are relatively long and it is possible to measure very apical effects in the cochlea. As mentioned, the permanent effect that was observed in previous investigations (18, 20) was not reproducible with the catheter in a first attempt. This might have been due to the lower steroid concentration, which seemed insufficient for a sustained effect.

A dose dependent action of triamcinolone on fibrosis was already reported in the early Sixties in cellophane tape stripped and therewith irritated skin on the forearm of volunteers (21). Interestingly, higher concentrations of triamcinolone acetonide seem to not be advantageous over lower dosages for the intralesional treatment of alopecia areata (22). Comparing our low-dose with the high-dose results, the high-dose treatment resulted in a longer and greater activity of the drug in terms of impedance reduction. At first fitting, before and after electrical stimulation, there was a clear reduction in the impedances in the high-dose group compared to controls over all electrodes (C1–C12), as well as individually for the basal (C9–C12) and medial (C6–C8) contact groups. At this time, the effects of the low-dose triamcinolone treatment were not visible after 17–24 days when compared to the controls. Thus, the effect of the higher dosage lasted significantly longer than of the low dosage. However, a statistically significant decrease of the impedances was only observed up to contact 6 (i.e., the basal and mediobasal contacts). Although there was a decline in the high-dose group also at apical contacts, this reduction was not statistically significant. Contact 6 is located 16.3 mm from the proximal/basal beginning of the electrode but the drug was applied to a depth of 20 mm into the cochlea. This discrepancy could be explained by the fact that the apical part of the Flex28 electrode is very thin, soft, and flexible. Under certain circumstances, this causes only a very slight trauma, or a mild foreign body, so that an anti-fibrotic effect could not be detected here. In addition, the drug may not have been distributed sufficiently to the more apical regions. Distribution of drugs in the cochlea is not completely understood and is based on diffusion, flow or transport. Based on knowledge from mouse cochleae, especially the diffusion of drugs from the base to the apex may be influenced by clearance and by transport between the scalae (23, 24). However, the data obtained for cochlear pharmacokinetics are mainly based on animal experiments and on transtympanic but not intracochlear drug delivery (17, 23–25).

When regarding the impedance values of individual patients, one patient of the high dose steroid group have increased impedances values (higher than the control) 3 days after surgery. The increase of impedances may be contributed to the change of composition of the perilymph after application of the crystalloid steroid solution. The effect of triamcinolone that was achieved in the present study was temporary. Between the first fitting and the 3 month re-fitting appointment, the impedances also increased in the high-dose group. Here, the effect of the crystalloid-applied steroid has faded as indicated by the rising impedances. Whether a putative foreign body reaction on the electrode array and subsequent inflammation has probably lead to formation of fibrotic tissue and rise of impedances is not proven yet but it is assumed. The role of material and surface properties on foreign body reactions and impedances is not fully understood and needs further investigation. However, hearing preservation in the lower frequencies is achieved even by the use of longer electrodes from the same manufacturer that has been used in the present study (26). Therefore, it seems unlikely that the electrode array has elicited a strong foreign body reaction. In humans, macrophages have been shown to establish direct physical contacts with both vestibular and cochlear axons and ganglion cell bodies in humans (27) and high-dose steroids suppress macrophage and progenitor cell proliferation in the brain (28). It could therefore be that a re-activation of macrophages after cessation of the application of high-dose steroids could lead to the rise of impedances to the level of the untreated controls. However, this is highly speculative and needs further investigation. After the decline of the effects of both high- and low-dose steroids, the impedances were similar among the groups and to the control group including the 1 year appointment. In order to achieve a permanent lowering of the impedances after implantation of Flex electrodes, a one-time, although high-dose and crystalloid application, of steroids seems therefore insufficient. This may be different for other electrode types and for selected patient groups. In addition, the immunological predisposition of each individual may influence the responsiveness to steroids and other anti-inflammatory agents. The initial immunological status before implantation can now be detected in tiny perilymph samples with significant variations between the patients (29). In the future, it may be possible to diagnose just these constellations in which steroid injections are most likely beneficial for the patient. Moreover, pump systems or even steroid-releasing electrodes for repeated or sustained application could be more successful if effective drug-levels can be sustained for a longer period inside the cochlea.

After cochlear implantation, low impedance values are assumed to be beneficial for the patients for several reasons. On the one hand, the impedances increase, the more scar tissue has formed around the electrode, acting as an isolation of the electric contacts (3). The less traumatic the insertion and the milder the immunological foreign body reaction afterwards, the less scar should be formed around the electrode array and hence the lower the impedances should be measurable (2, 3). Low impedances can therefore be used as a measure of low mechanical and immunological intra-cochlear trauma following CI provision (2, 3, 9). Furthermore, increased resistance leads to higher current requirements to achieve the same stimulation, possibly leading to higher power consumption (18, 20). In addition, it is also conceivable that an increased spread of excitation and thereby reduced channel separation may be caused by the scar tissue around the electrode contacts (18, 20).

In any case, our study demonstrates that the catheter can be safely inserted into deeper regions of the cochlea. We therefore assume that with this device, drugs can be applied up to 20 mm deep in the cochlea. Whether the drugs reach the apical region of the cochlea needs further investigation, e.g., by monitoring the influence of such a steroid application on the preservation of low frequency residual hearing. This is focus of current studies. Additionally, a protective effect of the treatment on the neuronal health in the cochlea seems mandatory for the performance independently of impedance values. In the clinical routine, however, there are still no reliable diagnostic tools to assess such effects.

The present data are naturally subject to some weaknesses. For example, the individual concentration of the drug in the cochlea cannot be detected and considerable differences may exist between the individuals. Furthermore, it is also not known and not measurable when the drug is cleared from the cochlea or when it is metabolized. Additionally, the groups with *n* = 5 were quite small. Whether increased impedances taken alone, however, worsen the outcome, for example speech comprehension, or minimize patient satisfaction has not yet been answered (30). The study design might also influence the retrieved results. Despite the fact that all patients (study groups with high and low dose steroids and control group) were implanted with the same electrode type, there were differences between the groups (i.e., age, gender, etiology of hearing loss, individual cochlear anatomy and expertise of the surgeon) that were not controlled. Thus, due to the multiple factors that influence these results, blinded multicentre studies with significantly larger patient groups are necessary to rule out confounding factors.

To conclude, with a high-dose, single application of triamcinolone using a cochlear catheter prior to the insertion of a Flex28 electrode, the impedances can be significantly reduced up to and including the first fitting, which took part on average 39.53 days postop (SD +/– 5.91 days). This effect was only significant in the basal and medial contact groups and lasted longer than with the lower dosage but is as well not permanent.

## Data Availability Statement

The raw data supporting the conclusions of this article will be made available by the authors, without undue reservation, to any qualified researcher.

## Ethics Statement

The studies involving human participants were reviewed and approved by Ethics Committee, Hannover Medical School, reference numbers 2740-2015 and 3279-2016. The patients/participants provided their written informed consent to participate in this study.

## Author Contributions

NP and AW: substantial contributions to the conception or design of the work, the acquisition, analysis and interpretation of data for the work, drafting the work, provided approval for publication of the content, and agreed to be accountable for all aspects of the work in ensuring that questions related to the accuracy or integrity of any part of the work are appropriately investigated and resolved. RS and LG: substantial contributions to the analysis and interpretation of data for the work, provided approval for publication of the content, and agreed to be accountable for all aspects of the work in ensuring that questions related to the accuracy or integrity of any part of the work are appropriately investigated and resolved. TL: substantial contributions to the conception or design of the work, revising the manuscript critically for important intellectual content, provided approval for publication of the content, and agreed to be accountable for all aspects of the work in ensuring that questions related to the accuracy or integrity of any part of the work are appropriately investigated and resolved.

### Conflict of Interest

The authors declare that the research was conducted in the absence of any commercial or financial relationships that could be construed as a potential conflict of interest.
